# A Novel Method of Multi-Information Acquisition for Electromagnetic Flow Meters

**DOI:** 10.3390/s16010025

**Published:** 2015-12-26

**Authors:** Wenhua Cui, Bin Li, Jie Chen, Xinwei Li

**Affiliations:** School of Mechatronic Engineering and Automation, Shanghai University, Shanghai 200072, China; jane.chen@shu.edu.cn (J.C.); LiXinwei@shu.edu.cn (X.L.)

**Keywords:** electromagnetic flow meter, multi-information, electrical excitation, impedance spectroscopy, multi-frequency synchronous signal, photovoltaic cell

## Abstract

In this paper, a novel method is proposed for multi-information acquisition from the electromagnetic flow meter, using magnetic excitation to measure the fluid velocity and electrochemistry impedance spectroscopy (EIS) for both the fluid quality and the contamination level of the transducer. The impedance spectra of the transducer are measured with an additional electrical stimulus in series with the electrode measurement loop. The series connection mode instead of the parallel one improves the signal-to-noise ratio (SNR) of the fluid velocity measurement and offers a wide range of impedance measurements by using a sample capacitance. In addition, a multi-frequency synchronous excitation source is synthesized based on the method of dual-base power sequences for fast EIS measurement. The conductivity measurements in the range of 1.7 μS/cm–2 mS/cm showed a relatively high accuracy with a measurement error of 5%, and the electrode adhesion detection on both with coating and no coating showed the ability of the qualitative determination of the electrode adhesion, which validated the feasibility of the multi-information acquisition method for the electromagnetic flow meter (EMFM).

## 1. Introduction

With the development of industry, the traditional electromagnetic flow meter (EMFM) only for fluid-velocity measurement could not meet the growing demand for monitoring both the fluid quality (e.g., fluid conductivity) and the contamination level of the transducer (e.g., electrode adhesion, empty pipe, *etc*.) [1] in real time. Generally, the fluid information is integrated from multiple sensors [2,3], and the contamination level of the transducer is inspected with much manpower, which would result in poor real-time performance, low accuracy and high maintenance cost. Due to these facts, a high-efficiency method, not at the cost of multiple sensors and much manpower, is desirable for the EMFM to obtain multiple types of information on both the fluid and the transducer [4,5,6].

A promising tool is to measure the wetted electrode impedance of the EMFM with an additional electrical stimulus. This method takes full advantage of the sensing electrodes in direct contact with the conductive fluid, and the internal information of the transducer could be obtained *in situ* without installation of other sensors. Some papers and patents have been published on two aspects, including the electrical excitation method and the parameter extraction theory [7], in the recent decade. The works in [8,9] introduce an approach to monitor the change of the fluid conductivity and the electrode adhesion with impedance measurement at only one frequency. However, the transducer of the EMFM is a complicated system, whose impedance is influenced by many factors, such as fluid, electrode adhesion, temperature, *etc*., and changes with the frequency. The measured single-frequency impedance could only be used for a rough evaluation. The work in [5] describes an exploration of the process diagnosis in the EMFM with the electrochemistry impedance spectroscopy (EIS) technique. However, it gives out only a preliminary result of 20% error for the measurement of medium conductivity in the range of about 25 μS/cm–4 mS/cm. The work in [10] presents an idea to detect the conductivity and electrode adhesion with multi-frequency impedance spectroscopy measurement. In all of the above methods, it should be noted that the additional electrical excitation source, is in parallel connection with the electrode measurement loop (called “parallel electrical excitation mode” in this paper) [11,12]. Because the electrical excitation source has the same ground with the instrumentation amplifier (INA), the equivalent input impedance of the INA would be reduced, leading to a further attenuation of the weak fluid-velocity signal and a deterioration of the SNR. Some product guides have stated to require the fluid conductivity to be higher than 10 μS/cm due to the imported electrical excitation source [13].

On the basis of our previous work [14], this paper introduces a novel method for the EMFM to acquire more valuable information with an additional electrical excitation. The electrical excitation is in series with the electrode measurement loop of the EMFM (called “serial electrical excitation mode” in this paper), overcoming the drawback of fluid signal attenuation in the parallel electrical excitation mode; a sample capacitance, identical to an open circuit for the sufficient low frequency during magnetic excitation, ensures a wide impedance measurement range in the period of electrical excitation; a multi-frequency synchronous (MFS) excitation source based on a photovoltaic (PV) cell [15], synthesized with the dual-base power sequences (DBPS) method proposed by the author in a previous paper [16], is employed for a fast impedance spectroscopy measurement. Furthermore, the electrical excitation module is electrically isolated from the electrode measurement loop by using a photoelectric-coupling control method.

The remainder of this paper is organized as follows. In Section 2, an equivalent model of the transducer is presented at first. The fluid-velocity signal loss under different modes of electrical excitation is then discussed. After that, a dual-excitation EMFM based on a PV cell is introduced, and the measurement principles of both the fluid-velocity and the electrode impedance spectroscopy are introduced. In Section 3, a brief description is given of the lab apparatus for the EMFM to obtain multiple information based on the data acquisition (DAQ) module and LabVIEW program. Additionally, the multi-frequency synchronous excitation signal based on the DBPS method is also introduced. In Section 4, two experiments, including medium conductivity identification and electrode adhesion detection, were carried out, and the experimental results are discussed.

## 2. Detecting Mechanism

### 2.1. Model of the Transducer

In the conventional EMFM, a pair of sensing electrodes are directly immersed in the conductive fluid, which would result in the formation of electrode-electrolyte interfaces. Since the conductive fluid moves in a direction perpendicular to the magnetic field with some velocity, induced voltage would be generated between the electrodes. With the effect of the Lorentz force, charged ions in the solution are attracted in the electrode-electrolyte interface [17,18,19]. This phenomenon is usually called the electrical double layer (EDL), the impedance of which could be described by a constant phase element (CPE) as follows:

(1) $$Z_{CPE}=\frac{1}{Q{\left(s\right)}^{n}}=\frac{1}{Q{\left(j\omega \right)}^{n}}$$

where the exponent *n* is between 0.5 and 1.0, with 0.5 corresponding to the case of a Warburg impedance, a charge diffusion dominated element, and 1.0 to a pure capacitance element.

**Figure 1:**
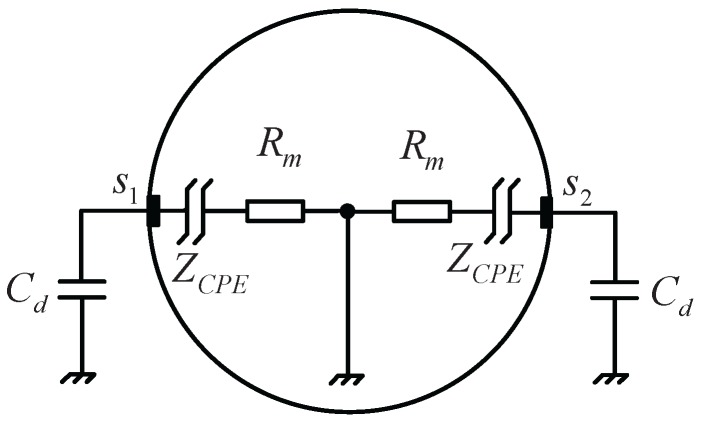
Equivalent circuit model of the transducer full of fluid.

Some contributions have been made on the model of the transducer [5,7]. An equivalent circuit that models the transducer full of fluid is illustrated in Figure 1. The impedance (denoted “$Z_{x}$”) between the electrode ($s_{1}$ or $s_{2}$) and the ground comprises a parallel connection of a capacitor $C_{d}$ representing the distributed capacitance between the electrode and the ground, a resistance $R_{m}$ representing the solution resistance and a constant phase element (CPE) $Z_{cpe}$, which describes the behavior of the EDL, wherein the latter are connected in series with each other. Then, the impedance $Z_{x}$ could be represented as:

(2) $$Z_{x}\left(R_{m},Q,n,C_{d},\omega \right)=\frac{{\left(Qs^{n}\right)}^{-1}+R_{m}}{1+\left({\left(Qs^{n}\right)}^{-1}+R_{m}\right)C_{d}s}$$

**Figure 2:**
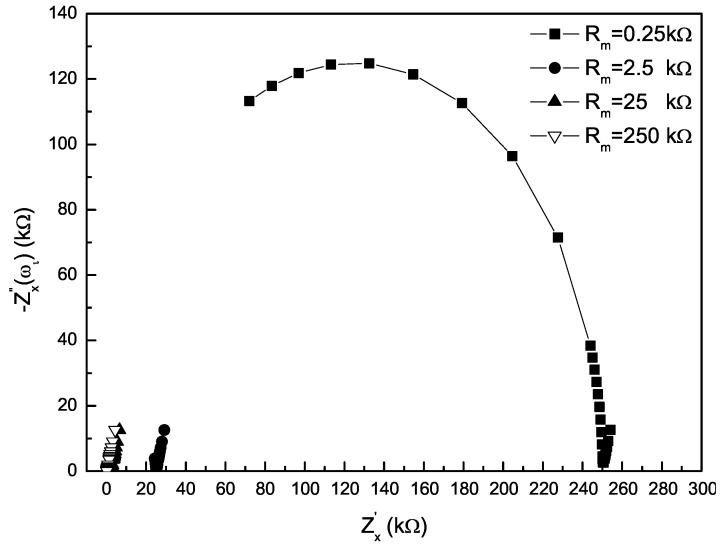
Nyquist plot of the impedance $Z_{x}$ for known parameters.

Consider some values of $R_{m}$ in the range of 0.25 up to 250 kΩ, which corresponds to the solution conductivity from 2000 down to 2 μS/cm, $Q=10^{-5}\left(S\cdot cm^{-2}\cdot s^{-n}\right)$, *n* = 0.8, $C_{d}$ = 100 pF representing a short signal line. According to Equation (2), the Nyquist plot of the impedance $Z_{x}$ over a frequency range from 2 Hz–10 kHz (restricted from the limited practical resources) is shown in Figure 2. In the low frequency range, the impedance $Z_{x}$ appears as a line of a certain slope, mainly dependent on the EDL impedance $Z_{cpe}$. Yet, in the intermediate frequency range, $Z_{x}$ is mostly determined by the solution resistance $R_{m}$, especially as the reactance of $Z_{x}$ is close to zero. In the relatively high frequency range, $Z_{x}$, contributed mainly by the capacitance $C_{d}$ and the solution resistance $R_{m}$, appears as a semicircle at the condition of $R_{m}$ large enough.

Thus, the impedance $Z_{x}$ contains the properties of the EMFM. By using the electrical impedance spectroscopy technique, the related valuable parameters could be extracted from the impedance spectra of the transducer.

### 2.2. Fluid-Velocity Signal Loss under Different Modes of Electrical Excitation

**Figure 3:**
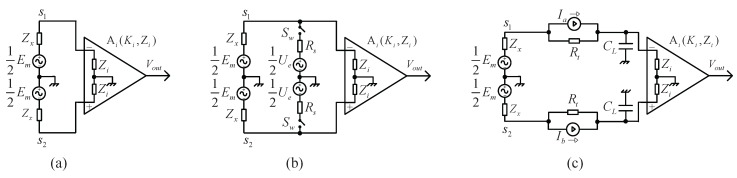
Principal diagram of the electrode measurement loop of the electromagnetic flow meter (EMFM). (**a**) No electrical excitation; (**b**) parallel electrical excitation; mode (**c**) serial electrical excitation mode.

Without electrical stimulus, the electrode measurement loop of the EMFM is illustrated schematically in Figure 3a. It consists of flow-induced voltage $E_{m}$, electrode impedance $Z_{x}$ and an INA, whose input impedance is $Z_{i}$ and amplification coefficient is $K_{i}$. Deduced from Ohm’s law, the differential voltage of the INA’s input signal is given by:

(3) $$V_{in-n}=\frac{Z_{i}}{Z_{x}+Z_{i}}\times E_{m}$$

To acquire the impedance spectrum of $Z_{x}$, there must be another known stimulus fed into the transducer via the sensing electrodes. As shown in Figure 3b, a common method is to import the electrical excitation in parallel connection mode, wherein the electrical excitation module, consisting of an excitation voltage source and a sample resistance $R_{s}$, is in parallel connection with the input of the INA via an analog switch $S_{w}$. Supposing the internal impedance of the voltage source $U_{e}$ is zero, the impedance of the excitation module could be written as $Z_{l}=R_{s}+Z_{w}$, with $Z_{w}$ denoting the switch impedance. Since magnetic excitation alternates with electrical excitation in the time domain in this research, $U_{e}$=0 in the period of magnetic excitation. Then, the differential voltage of the INA’s input signal $V_{in-p}$ is:

(4) $$V_{in-p}=\frac{Z_{i}}{Z_{x}+Z_{i}}\times E_{m}\times {\left(1+\frac{Z_{x}}{\left(1+Z_{x}/Z_{i}\right)Z_{l}}\right)}^{-1}$$

From Equation (3) and Equation (4), the fluid-velocity signal loss percentage in the parallel electrical excitation mode could be calculated as:

(5) $$p_{loss}\left(\%\right)=\frac{V_{in-n}-V_{in-p}}{V_{in-n}}=\frac{Z_{x}}{Z_{x}+\left(1+Z_{x}/Z_{i}\right)Z_{l}}\times 100\left(\%\right)$$

Generally, $Z_{i}$ is of the order of magnitude of $10^{9}\;\Omega$, and that of $Z_{x}$ would not be larger than $10^{6}\;\Omega$, even at the condition of serious electrode adhesion. Moreover, $Z_{l}$ is equivalent to $Z_{w}$ during magnetic excitation, due to the fact that $Z_{w}$ under off-state is of the order of magnitude of $10^{7}\;\Omega$ to $10^{8}\;\Omega$, much larger than $R_{s}$. Thus, Equation (5) becomes:

(6) $$p_{loss}\left(\%\right)=\frac{Z_{x}}{Z_{x}+Z_{l}}\times 100\%$$

Consider the impedance $Z_{x}$ in cases such as low medium conductivity or serious electrode contamination. The related fluid-velocity signal loss percentages are summarized in Table 1. Supposing the value of $Z_{x}$=0.1 MΩ corresponding to the medium conductivity of 10 μS/cm, $p_{loss}$ is calculated for some values of $Z_{x}$ in the range from 0.1–10 MΩ at the conditions of $Z_{l}$ = 10 MΩ, 30 MΩ, 100 MΩ. If $Z_{l}$ = 10 MΩ, $p_{loss}$= 0.99% at $Z_{x}$ = 100 kΩ; $p_{loss}$ = 9.08% at $Z_{x}$ = 1 MΩ; $p_{loss}$ = 49.75% at $Z_{x}$ = 10 MΩ. If $Z_{l}$ = 100 MΩ, $p_{loss}$ = 0.1% at $Z_{x}$ = 100 kΩ; $p_{loss}$ = 0.99% at $Z_{x}$ = 1 MΩ; $p_{loss}$ = 9% at $Z_{x}$ = 10 MΩ. In addition, to obtain a wide measurement range, several sample resistors with resistances of different orders of magnitude are required to be connected in parallel to match the measured impedance $Z_{x}$, making the fluid signal loss more serious.

**Table 1:** Fluid-velocity signal loss in the parallel electrical excitation mode.

$Z_{x}\;\left(M\Omega \right)$	$p_{loss}$		
	$Z_{l}=10\;M\Omega$	$Z_{l}=30\;M\Omega$	$Z_{l}=100\;M\Omega$
0.1	0.99%	0.33%	0.1%
1	9.08%	3.22%	0.99%
10	49.75%	24.81%	9%

The electrode measurement loop of the EMFM in the serial electrical excitation mode proposed in this paper, is illustrated schematically in Figure 3c. The electrical excitation module, consisting of a current source $I_{a}$ (and $I_{b}$) and a resistance $R_{t}$, serving as a current-to-voltage converter ($R_{t}$=3.6 kΩ), is in series between the sensing electrode and the input of the INA. Moreover, there is a pair of sample capacitances $C_{l}$ connected to the inputs of the INA. During magnetic excitation, the excitation source with zero current ($I_{a}=0$ and $I_{b}=0$) is identical to an ideal open circuit. On the basis of transient circuit analysis, the differential voltage of the INA’s input signal $V_{in-s}$ is:

(7) $$V_{in-s}=\frac{Z_{i}}{Z_{x}+R_{t}+Z_{i}}\times E_{m}\times \left(1-e^{-t/\tau }\right)$$

where $\tau =\left(Z_{x}+R_{t}\right)\times C_{l}$. Having $R_{t}\ll Z_{i}$, so Equation (7) becomes:

(8) $$V_{in-s}=\frac{Z_{i}}{Z_{x}+Z_{i}}\times E_{m}\times \left(1-e^{-t/\tau }\right)$$

By Equation (8), provided that the time interval between the magnetic excitation starting and the fluid-velocity signal sampling is larger than 5τ, the fluid-velocity signal loss percentage would be smaller than the value of $e^{-5}=0.67\%$. Table 2 illustrates the related time intervals (5τ) to some values of $Z_{x}$ in the range from 0.1–10 MΩ at the conditions of $C_{l}$ = 1 nF, 10 nF, 100 nF. For most applications, such as medium conductivity measurement, electrode adhesion evaluation and empty pipe detection, the capacitance $C_{l}$ would be selected with a value equal or smaller than 10 nF. If $C_{l}$ = 10 nF, 5τ = 50 ms at $Z_{x}$ = 1 MΩ; 5τ = 500 ms at $Z_{x}$ = 10 MΩ. The above time intervals are feasible for the reason that the magnetic excitation cycle of the EMFM is usually 16 or 32 multiples of the power frequency cycle (20 ms in a 50-Hz system). Nevertheless, for special applications, such as the property detection of the electrode adhesion, to obtain the parameters of the EDL, the capacitance of $C_{l}$ is suggested to be larger than 10 nF. At this time, even when the electrode is seriously contaminated (not fully insulated), the signal loss still could be small.

**Table 2:** Suitable time intervals in the serial electrical excitation mode.

$Z_{x}\;\left(M\Omega \right)$	5τ(ms)		
	$C_{l}$ = 1 nF	$C_{l}$ = 10 nF	$C_{l}$ = 100 nF
0.1	0.52	5.2	52
1	5	50	500
10	50	500	5000

Therefore, if the condition t≥5τ (t denotes the time interval) was fulfilled, the conventional flow-rate measurement equation [20] would be also available in the serial electrical excitation mode, given by:

(9) $$V_{O}=K_{i}\times \frac{Z_{i}}{Z_{x}+Z_{i}}\times E_{m}=B\times D\times \nu$$

where *B* is the magnetic flux density, *D* is the diameter of the tube in meters and *ν* is the flow velocity.

### 2.3. EIS Measurement Principle in Serial Electrical Excitation Mode

**Figure 4:**
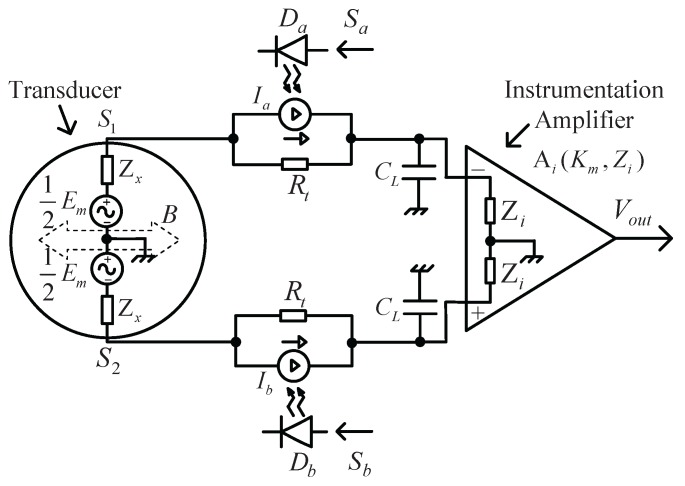
Schematic diagram of the dual-excitation EMFM based on a PV cell.

The schematic diagram of a dual-excitation EMFM based on the PV cell is shown in Figure 4. It consists of a pair of sensing electrodes ($s_{1}$ and $s_{2}$), a pair of specially-designed PV converters, a pair of sample capacitances $C_{l}$ and an INA. Each PV converter consists of a PV cell and a resistor $R_{t}$ (a current-to-voltage converter) in parallel connection. During electrical excitation, $E_{m}$ = 0. The PV cell ($PVC_{a}$ or $PVC_{b}$), activated by an adjacent LED($D_{a}$ or $D_{b}$), which is driven by a controllable AC current ($S_{a}$ or $S_{b}$), generates the photovoltaic current ($I_{a}$ or $I_{b}$) based on the photovoltaic effect. The current signal for electrical excitation could be expressed mathematically as:

(10) $$\dot{I_{I}}=\dot{I_{a}}+\dot{I_{b}}$$

With the resistor $R_{t}$ converting current to voltage, the terminal voltages of the two PV cells are $E_{a}$ and $E_{b}$, respectively. Then, the voltage signal for electrical excitation becomes:

(11) $$\dot{V_{I}}=\dot{E_{a}}+\dot{E_{b}}$$

Thus, the differential voltage of the INA’s input signal could be calculated as:

(12) $$\dot{V_{O}}=\frac{Z_{C_{l}}}{Z_{x}+Z_{C_{l}}}\times \dot{V_{I}}$$

where $Z_{C_{l}}$ is the impedance of the sample capacitance $C_{l}$. Since the signal $I_{I}$ generated for electrical excitation is a periodic odd signal, according to the Fourier series theory, $V_{I}$ could be represented as an infinite summation of sinusoidal harmonics.

(13) $$\dot{V_{I}}=\sum_{i=1}^{\infty }A_{i}sin\left(\omega_{i}t+\alpha_{i}\right)$$

where $A_{i}$ and $\alpha_{i}$ are the magnitude and phase of the *i*-th harmonic, respectively.

At the same time, the resulting signal $V_{O}$ also could be expressed as an infinite summation of sinusoidal harmonics.

(14) $$\dot{V_{O}}=\sum_{i=1}^{\infty }B_{i}sin\left(\omega_{i}t+\beta_{i}\right)$$

where $B_{i}$ and $\beta_{i}$ are the magnitude and phase of the *i*-th harmonic, respectively. Therefore, substituting Equation (13) and Equation (14) into Equation (12), solving for $Z_{x}$ yields:

(15) $$Z_{x}\left(\omega_{i}\right)=Z_{x}{\left(\omega_{i}\right)}^{\prime }-jZ_{x}{\left(\omega_{i}\right)}^{\prime \prime }=\frac{A_{i}}{B_{i}}\times \frac{sin\theta_{i}}{C_{l}\omega_{i}}-j\left(\frac{A_{i}}{B_{i}}\times \frac{cos\theta_{i}}{C_{l}\omega_{i}}-\frac{1}{C_{l}\omega_{i}}\right)$$

where $Z_{x}\left(\omega_{i}\right)$ is the impedance $Z_{x}$ at the frequency $f_{i}$, $\theta_{i}=\alpha_{i}-\beta_{i}$. Then, from Equation (15), the amplitude and phase at the frequency $f_{i}$ could be written as:

(16) $$\left\{\begin{array}{c} \left|Z_{x}\left(\omega_{i}\right)\right|=\sqrt{{\left(Z_{x}{\left(\omega_{i}\right)}^{\prime }\right)}^{2}+{\left(Z_{x}{\left(\omega_{i}\right)}^{\prime \prime }\right)}^{2}}\\ ∠Z_{x}\left(\omega_{i}\right)=-arctan\left(Z_{x}{\left(\omega_{i}\right)}^{\prime \prime }/Z_{x}{\left(\omega_{i}\right)}^{\prime }\right) \end{array}\right.$$

## 3. System Realization

**Figure 5:**
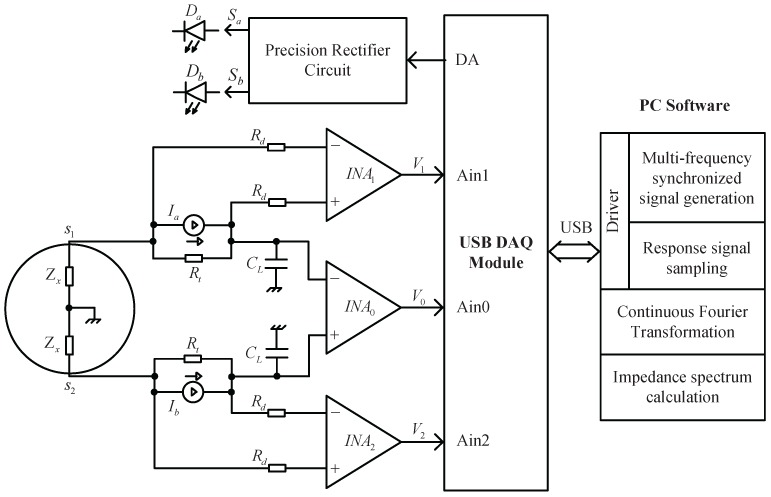
Waveform of the dual-base power sequence (DBPS) signal F(2, 3, $\omega_{0}$, t) in one period.

After being amplified by the INA, both the fluid-velocity signal and the impedance signal will be processed with their respective circuits, since the signal amplitudes are not on the same order of magnitude. Figure 5 shows the block diagram of the EIS measurement system for the EMFM. It consists of the electrode measurement loop, a DAQ module and a LabVIEW program on a PC (personal computer). The signal processing for EIS measurement consists of two main parts: one is to generate a MFS excitation signal $I_{I}$; the other is to sample the resulting signals $E_{a}$, $E_{b}$ and $V_{O}$. The three response signals, output from the respective amplifiers (INA${}_{0}$, INA${}_{1}$ and INA${}_{2}$), are sampled simultaneously by the analog-to-digital converter located in the DAQ module. Next, the sampled signals would be sent to the PC via the USB cable and then processed by the LabVIEW program with the FFT technique. Finally, the impedance $Z_{x}\left(\omega_{i}\right)$ would be calculated according to Equation (15).

For a fast impedance spectroscopy measurement, the DBPS method [16] is proposed for the synthesis purpose of the MFS signal. In this method, to make an M × N-frequency DBPS signal F(M, N, $\omega_{0}$, t), M groups, each with N odd square waves, are applied simultaneously with a matrix of weight coefficients. The frequencies of all members of a group increase by powers of three, whilst the frequencies of the *n*-th (0 ≤ *n* ≤N−1) member of each group could be constructed as a geometric sequence with a common ratio of two. The synthesized signal offers advantages, such as a wide frequency spectrum, a flat amplitude spectrum, simultaneous phase, high energy efficiency, *etc.* For simplicity, let M = 2, N = 3; the six-frequency signal F(2, 3, $\omega_{0}$, t), containing primary harmonics at frequencies of $f_{0}$, 2$f_{0}$, 3$f_{0}$, 6$f_{0}$, 9$f_{0}$ and 18$f_{0}$, could be expressed mathematically as:

(17) $$F\left(2,3,\omega_{0},t\right)=E\times \sum_{m=0}^{1}\left[2Sgn\left(sin\left(2^{m}\omega_{0}t\right)\right)+2\sum_{n=1}^{2}Sgn\left(sin\left(2^{m}3^{n}\omega_{0}t\right)\right)\right]$$

where *E* is the amplitude, $\omega_{0}$ is the fundamental angular frequency and Sgn(x) is the signum function.

(18) $$Sgn\left(x\right)=\left\{\begin{array}{cc} 1 & for\;x>0\\ 0 & for\;x=0\\ -1 & for\;x<0 \end{array}\right.$$

Provided E = 1, six sequence functions from 3Sgn(sin($\omega_{0}$t)) to 2Sgn(sin(18$\omega_{0}$t)) are applied to compose F(2, 3, $\omega_{0}$, t) according to Equation (17). Since the highest frequency of the sequence functions is 18$f_{0}$, the period $T_{0}$ of F(2, 3, $\omega_{0}$, t) could be divided into 36 units; the resultant vector could be written as:

(19) $$\begin{array}{c} F\left(2,3,\omega_{0},t\right)=[14,10,10,2,10,6,6,2,10,-4,-4,-8,8,4,4,-4,4,0,0,-4,4,\\ -4,-4,-8,8,4,4,-10,-2,-6,-6,-10,-2,-10,-10,-14] \end{array}$$

**Figure 6:**
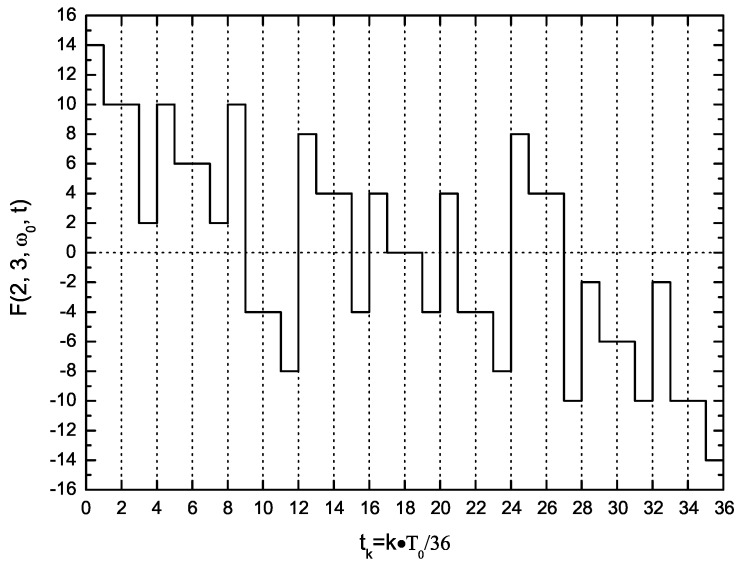
Waveform of the DBPS signal F(2,3,$\omega_{0}$,t) in one period.

The time-domain waveform of F(2, 3, $\omega_{0}$, t) is shown in Figure 6.

The sequence elements of the DBPS signal F(2, 3, $\omega_{0}$, t) in one period would be constructed with the LabVIEW program on the computer and transmitted to the DAQ module along with the frequency $f_{clk}=36f_{0}$ via the USB cable. The D/A converter located on the DAQ module would circularly produce every element with the frequency $f_{clk}$ and output via the terminal DA (output port of the DAQ module depicted in Figure 5). Thus, a voltage signal of F(2, 3, $\omega_{0}$, t) is generated and then would be split into positive and negative ($S_{a}$ and $S_{b}$) via a precision rectifier circuit, to drive the LEDs of both Da and Db, respectively. Based on the photovoltaic effect, the PV cells would produce proportional induced currents $I_{a}$ and $I_{b}$, which exactly constitutes the excitation current $I_{I}$. The frequency of the DBPS signal for the EIS measurement could be adjusted via the frequency $f_{clk}$; e.g., if $f_{clk}$ = 360 Hz was set, the primary harmonic frequencies of the DBPS signal would be in the range of 10–180 Hz; If $f_{clk}$ = 36 kHz was set, they would be in the range of 1–18 kHz.

## 4. Experimental Results and Discussion

To validate the method of multi-information acquirement for the EMFM, two experiments, including medium conductivity identification and electrode adhesion detection, were carried out with stationary fluids. The EMFM used for experiments had a nominal diameter of 100 mm, and the signal line was 1 m long.

### 4.1. Medium Conductivity Identification

The conductivity identification experiments covered a conductivity range from 1.7 μS/cm–2.1 mS/cm. The electrolyte solutions for experiments were mixed with different portions of deionized water (conductivity of about 0.1 μS/cm, a resistance of about 10 MΩ) and KCl, at a temperature of about $25{\;}^{\circ }$C. Additionally, the reference conductivity values were provided by a commercial conductivity meter.

**Figure 7:**
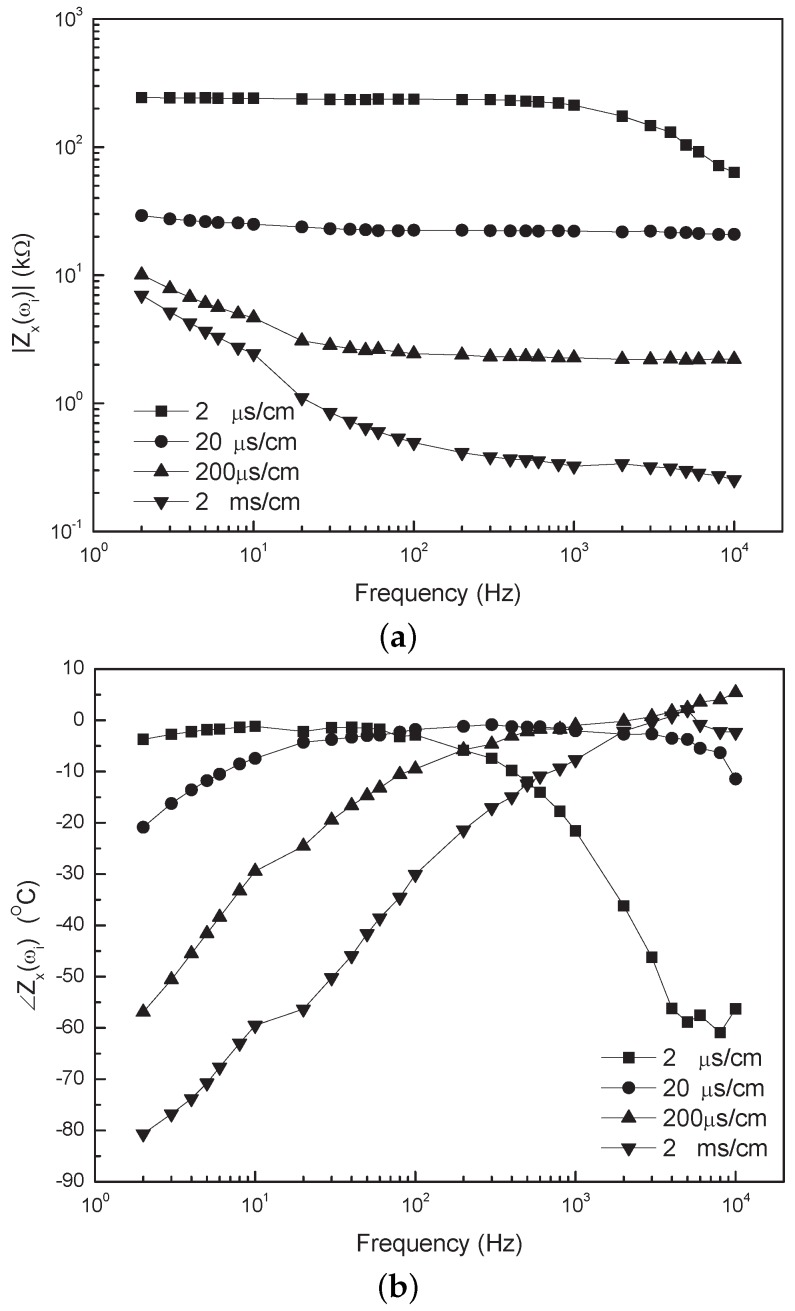
Bode plot of the measured impedance spectrum of the transducer for different solution conductivities. (**a**) Absolute value of impedance *vs*. frequency; (**b**) Phase of impedance *vs*. frequency.

**Figure 8:**
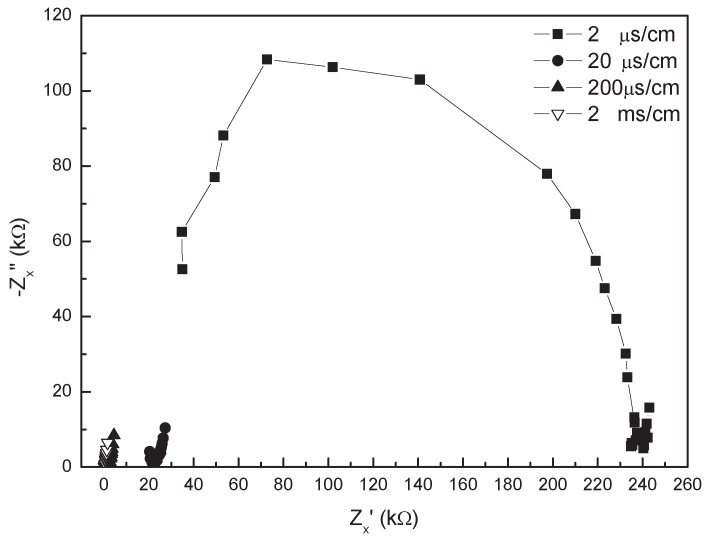
Nyquist plot of the measured impedance spectrum of the transducer for different solution conductivities.

The measured impedance spectrum of the transducer for different solution conductivities is illustrated in Figure 7 (Bode plot) and Figure 8 (Nyquist plot). The Nyquist plot is in agreement with the one shown in Figure 2, so that the model of the transducer introduced in Section 2.1 is feasible. In the low-frequency range of 2–100 Hz, the absolute value of impedance $\left|Z_{x}\left(\omega_{i}\right)\right|$ for the conductivities, such as 200 μS/cm and 2 mS/cm, have an obvious down-trend with the increase of the frequency in Figure 7a, which is shown as an oblique line in the Nyquist plot. The dominant effect of the linear portion comes from the EDL, and additionally, it would occur in a higher frequency range as the solution conductivity increases. In the intermediate-frequency range of about 0.1–1 kHz, the spectrum of $Z_{x}$ keeps flat for the conductivities in the range of 2 μS/cm–2 mS/cm in Figure 7a, and the related $-Z_{x}^{\prime \prime }\left(\omega_{i}\right)$ is close to zero in Nyquist plot. This is because $Z_{x}$ is mainly determined by the solution resistance at this moment. Especially, when $∠Z_{x}\left(\omega_{i}\right)$ is zero, $\left|Z_{x}\left(\omega_{i}\right)\right|$ equals the solution resistance. In the high-frequency range (>1 kHz), $\left|Z_{x}\left(\omega_{i}\right)\right|$ for the conductivity of 2 μS/cm decreases with increasing the frequency, which is shown as a semicircular arc in the Nyquist plot. According to the EIS theory, the arc portion is mainly caused by a resistor parallel with a capacitance. In our cases, this is caused by the solution resistance and the distributed capacitance of the signal line, because the effect of the dielectric constant of the fluid would become visible only at much higher frequencies. Whilst for the solution conductivity equal or higher than 200 μS/cm, the arc portion disappears because the solution resistance is so small that the effect of the distributed capacitance is not obvious in the considered frequency region.

**Figure 9:**
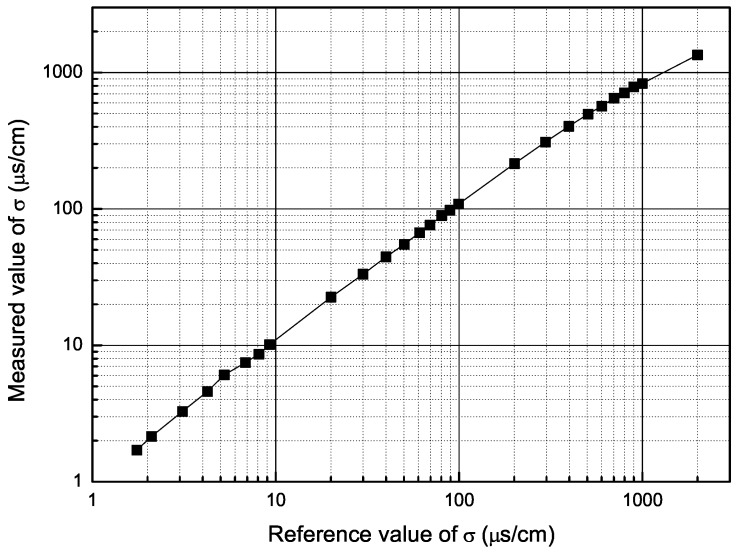
Measured value of the medium conductivity *vs*. the reference value.

According to the above analysis, the solution resistance $R_{m}$ nearly equals the absolute value of impedance $\left|Z_{x}\left(\omega_{i}\right)\right|$ at the condition of the related phase $∠Z_{x}\left(\omega_{i}\right)$ close to zero. The medium conductivity could be calculated by the formula $\sigma =k/(2R_{m})$, where *k* is a constant associated with the electrode area and electrode spacing. The experimental results (in Figure 9) showed a linear relationship between the measured and reference conductivity. Limited by the measurement accuracy of the current system, the measurement error is within 5% for the conductivities in the range of 1.7 μS/cm–2 mS/cm, which is relatively satisfactory compared to the 20% for the conductivities in the range of about 25 μS/cm–4 mS/cm in the literature [5].

It should be stated that the above impedance spectroscopy was measured by using the traditional frequency-sweep approach. Nevertheless, in practical applications, the effective frequencies for conductivity identification mainly concentrate in the range of 0.1–1 kHz. Thus, the solution conductivity could be measured with one-time electrical excitation by using a 2 × 3-frequency DBS signal, which contains six frequencies in the range of 0.1–1.8 kHz.

### 4.2. Electrode Adhesion Detection

It is a little difficult to build a practical environment for the experiment of electrode adhesion detection. In this research, the experiment was carried out with four cases of soft cream, hard cream, chewing gum and MoS2. In each case, the electrode surface was cleaned at first and then fully covered with dirt. After that, the pipe was filled with tap water. In addition, for the purpose of comparative analysis, the impedance spectroscopy of the transducer full of tap water was also tested at the condition of no electrode fouled. The measured impedance data for both medium conductivity identification and electrode adhesion detection are analyzed using the Zsimpwin software. The parameters, such as $R_{m}$ (the solution resistance), *Q* and *n* (the parameters to describe the EDL) for different cases, obtained based on the model of the transducer defined by Equation (2), are listed in Table 3.

**Table 3:** Three parameters $R_{m}$, *Q* and *n* for different cases. Case 1: no electrode fouled; Case 2: both electrodes fouled.

Conditions		$R_{m}$	*Q*	*n*
		k(Ω)	$\left(S\cdot cm^{-2}\cdot s^{-n}\right)$	
Case 1	200 μS/cm	2.4	14.7 $\times \;10^{-6}$	0.8
	400 μS/cm	1.24	15.7 $\times \;10^{-6}$	0.81
	600 μS/cm	0.85	16 $\times \;10^{-6}$	0.81
	1 mS/cm	0.5	16.7 $\times \;10^{-6}$	0.75
	tap water	1.518	15 $\times \;10^{-6}$	0.78
Case 2	soft cream	0.946	26 $\times \;10^{-6}$	0.86
	hard cream	2.57	21 $\times \;10^{-6}$	0.8
	chewing gum	18	12 $\times \;10^{-6}$	0.63
	MoS2	0.007	5 $\times \;10^{-6}$	0.23

Obviously, for the case of no electrode fouled (Case 1), the parameter $R_{m}$ changes with conductivity, whereas the parameters *Q* and *n*, which denote the properties of the EDL, almost keep constant, such as *Q* in the range of about $14.7\times 10^{-6}$–$16.7\times 10^{-6}\;\left(S\cdot cm^{-2}\cdot s^{-n}\right)$, *n* of about 0.8. Yet, when the electrodes are fouled, all three parameters $R_{m}$, *Q* and *n*, change with the coating. Compared to the reference values of tap water ($Q=15\times 10^{-6}$, n=0.78), *Q* increases to $21\times 10^{-6}$, $26\times 10^{-6}$, when the electrodes are coated with adhesive materials of relatively high conductivity, such as soft cream, hard cream. Additionally, when the adhesive conductivity is a bit low, such as gum, MoS2, the related *Q* decreases to $12\times 10^{-6}$, $5\times 10^{-6}$, and the related *n* decreases to 0.63, 0.23. Thus, the parameters *Q* and *n* could be used to determine whether electrode contamination occurs and to evaluate the properties of the adhesive material qualitatively; e.g., when both *Q* and *n* change, the electrode of the transducer should have been contaminated. Furthermore, if *Q* and *n* get smaller, the adhesive conductivity would be relatively low, and the related electrode impedance $Z_{x}$ at the magnetic excitation frequency might be large enough to affect the fluid velocity signal measurement. If *Q* gets larger, the adhesive conductivity would be relatively high, and the related electrode impedance $Z_{x}$ would not be large enough to affect the fluid signal measurement. The relationship between the impedance $Z_{x}$ and the fluid-velocity signal, expressed as Equation (9), could be used to compensate the fluid signal loss caused by the change of $Z_{x}$, especially for the cases of the electrode seriously contaminated or the fluid with low conductivity.

It should be stated that, for the reason that the practical contamination is very much complicated, the above results obtained based on only one simple model are rough. This paper is only a primary work; bigger or better studies need to be carried out to investigate the electrode adhesion detection.

## 5. Conclusions

This research work mainly contributes a multi-information acquisition method for the EMFM. A dual-excitation electromagnetic flow meter based on PV cells was designed and implemented. With fluid velocity measured with magnetic excitation, more valuable parameters could be obtained based on the EIS measurement of the transducer, by applying an additional electrical excitation in series connection with the original electrode measurement loop and a sample capacitance. The flow-rate signal loss would be lower than 0.67%, so long as a time interval of 5τ is taken into account between the magnetic excitation starting and the fluid-velocity signal sampling. The multi-frequency synchronous excitation signal based on the DBPS method has features such as a wide frequency spectrum, flat amplitude spectrum, high efficiency, *etc.* Conductivity identification experiments were carried out for the solution conductivities in the range of 1.7 μS/cm–2 mS/cm, with measurement error within 5%. Based on the proposed transducer model, the properties of the electrode coating could be judged roughly.

Further research should focus on the electrode adhesion detection. Moreover, both the software and the hardware of this system are required to be improved for higher accuracy.
